# Acute Cytomegalovirus Colitis Presenting As Exacerbation of Ulcerative Colitis: A Case Report

**DOI:** 10.7759/cureus.54903

**Published:** 2024-02-25

**Authors:** Varun Daiya, Nishtha Manuja, Sourya Acharya, Sunil Kumar, Sharwari Jaiswal

**Affiliations:** 1 Medicine, Jawaharlal Nehru Medical College, Datta Meghe Institute of Higher Education & Research, Wardha, IND; 2 Dermatology, Jawaharlal Nehru Medical College, Datta Meghe Institute of Higher Education & Research, Wardha, IND

**Keywords:** individualized treatment, opportunistic infections, tofacitinib, immunosuppressive therapy, cytomegalovirus colitis, ulcerative colitis

## Abstract

This case report presents a 24-year-old female with a history of ulcerative colitis (UC) who sought care for symptoms initially suggestive of the disease exacerbation but was later diagnosed as acute cytomegalovirus (CMV) colitis. The patient's clinical course, marked by watery diarrhea, blood in stools, vomiting, and fever, raised suspicion of a UC flare. However, a nuanced diagnostic approach revealed CMV superinfection, including computed tomography, colonoscopy, and tissue polymerase chain reaction (PCR). The patient's immunosuppressive history, with prior treatment, including intravenous infliximab and azathioprine, contributed to CMV infection. Treatment involved initiation of tofacitinib and antiviral therapy with valganciclovir. This case underscores the diagnostic challenges in distinguishing between infectious complications and UC exacerbations, necessitating a tailored, multidisciplinary approach for optimal management. It highlights the delicate balance required when managing UC patients on immunosuppressive regimens, emphasizing the importance of timely diagnosis and individualized treatment strategies in complex clinical scenarios.

## Introduction

Ulcerative colitis (UC) is a chronic inflammatory bowel disease characterized by relapsing and remitting inflammation of the colonic mucosa. Despite advances in therapeutic approaches, managing UC remains challenging, often requiring immunosuppressive agents to control symptoms and prevent disease progression [1]. Patients with UC are susceptible to opportunistic infections, particularly when undergoing immunosuppressive therapies, making diagnosing and managing infectious complications crucial in the clinical setting [2]. One such opportunistic infection is cytomegalovirus (CMV) colitis, which can present with symptoms mimicking an exacerbation of UC. CMV, a member of the herpesvirus family, can infect immunocompromised individuals, leading to various clinical manifestations, including gastrointestinal involvement [3]. In patients with UC, distinguishing between an exacerbation of the underlying inflammatory bowel disease and superimposed CMV infection is essential for appropriate and timely intervention [4].

Immunosuppressive therapies, such as corticosteroids, biologics, and immunomodulators, further complicate the clinical picture by potentially contributing to CMV infection. The coexistence of UC and CMV colitis poses diagnostic challenges due to overlapping symptoms and endoscopic findings, necessitating a multidisciplinary approach for accurate diagnosis and effective management [5]. In this context, we present a case of a young female with a history of UC, initially presenting with symptoms suggestive of an exacerbation, which was later revealed to be acute CMV colitis. This case underscores the importance of considering opportunistic infections in the differential diagnosis of inflammatory bowel disease exacerbations and highlights the need for a comprehensive approach to diagnosis and management.

## Case presentation

A 24-year-old female sought care at the outpatient department of a tertiary care hospital, presenting with complaints of watery diarrhea, 8-10 episodes per day, with blood in stools persisting for the past five days. She also reported vomiting, loss of appetite, abdominal pain, and fever for the same duration. She had a previous hospitalization a year ago for similar symptoms when she was diagnosed with ulcerative colitis and treated with intravenous infliximab following the initial treatment, which included steroids, azathioprine, and later biologics. She completed the induction phase of biological treatment and received three maintenance doses of infliximab later, she was then shifted to tofacitinib, to which she responded. 

Upon physical examination, the patient appeared pale, icterus was absent, and tenderness over the lower abdomen was noticed on palpation. Laboratory tests under complete blood count revealed anemia with a hemoglobin level of 7.6 gm/dl and elevated C-reactive protein levels at 26.0 mg/L (Table 1).

**Table 1:** Laboratory results of the patient MCV - mean corpuscular volume; TLC - total leucocyte count; ALT - alanine transaminase; APTT - activated partial thromboplastin time; PT - prothrombin time; INR - international normalized ratio; CMV - cytomegalovirus; PCR - polymerase chain reaction Clostridium difficle toxin A and B - negative <0.13, equivocal 0.13 to 0.37, and positive >0.37

Lab parameters	Observed value	Normal range
Hemoglobin	8.1 g/dL	13-17 g/dL
MCV	89 fL	83-101 fL
TLC	5700 cells/cu mm	4,000-10,000 cells/cu mm
Platelets	2.60 lakh/ cu mm	1.5-4.1 lakh/ cu mm
Urea	20 mg/dL	19-43 mg/dL
Creatinine	1.1 mg/dL	0.66-1.25 mg/dL
Sodium	132 mmol/L	137-145 mmol/L
Potassium	3.4 mmol/L	3.5-5.1 mmol/L
Alkaline phosphatase	40 U/L	38-126 U/L
ALT	48 U/L	50 U/L
Aspartate aminotransferase	44 U/L	17-59U/L
Albumin	3.2 g/dL	3.5-5 g/dL
APTT	30 sec	29.5 sec
PT	20 sec	<20 sec
INR	1.1	1-1.5
Unconjugated bilirubin	0.2 mg/dl	0.0- 1.1 mg/dl
Conjugated bilirubin	0.1 mg/dl	0.0-0.3 mg/dl
Total bilirubin	0.3 mg/dl	0.2-1.3mg/dl
C reactive protein	26 mg/dl	1-6 mg/dl
Clostridium difficle toxin A and B	0.01	Negative <0.13
CMV DNA PCR tissue	Positive	

**Figure 1:**
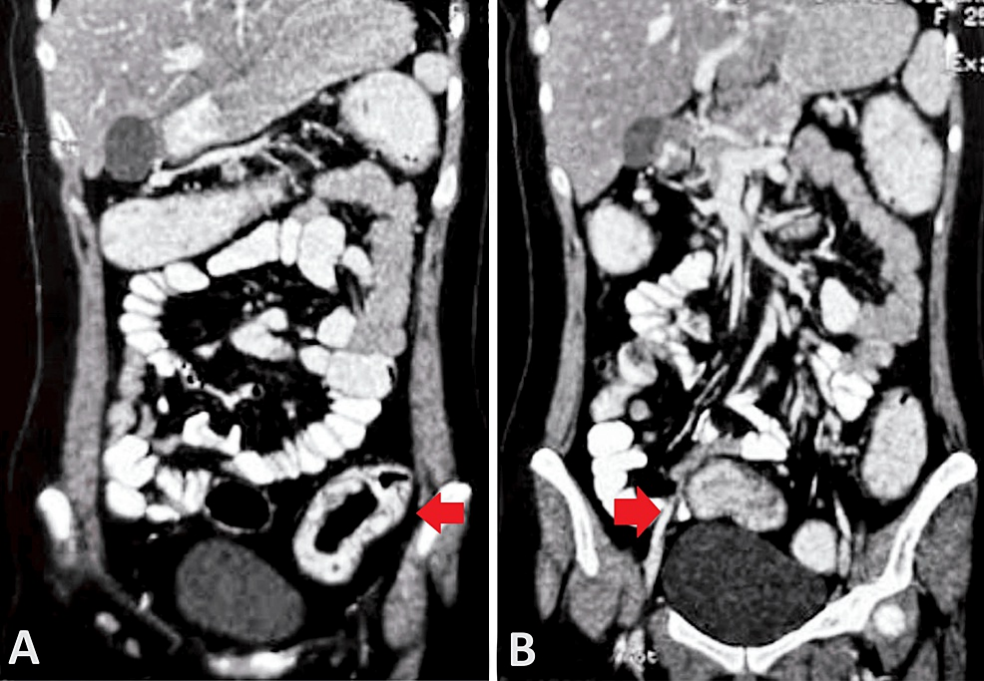
Contrast-enhanced computed tomography scan of abdomen and pelvis coronal sections (A) and (B) showing heterogeneously enhancing diffuse edematous wall thickening involving the rectosigmoid colon (red arrows)

Computed tomography of the abdomen and pelvis showed diffuse edematous wall thickening in the ascending colon, proximal part of the transverse colon, and rectosigmoid colon, along with enlarged perirectal and mesenteric lymph nodes suggestive of an infective and inflammatory etiology (Figure 1). Moderate fatty infiltration of the liver and mild splenomegaly was also noted.

**Figure 2:**
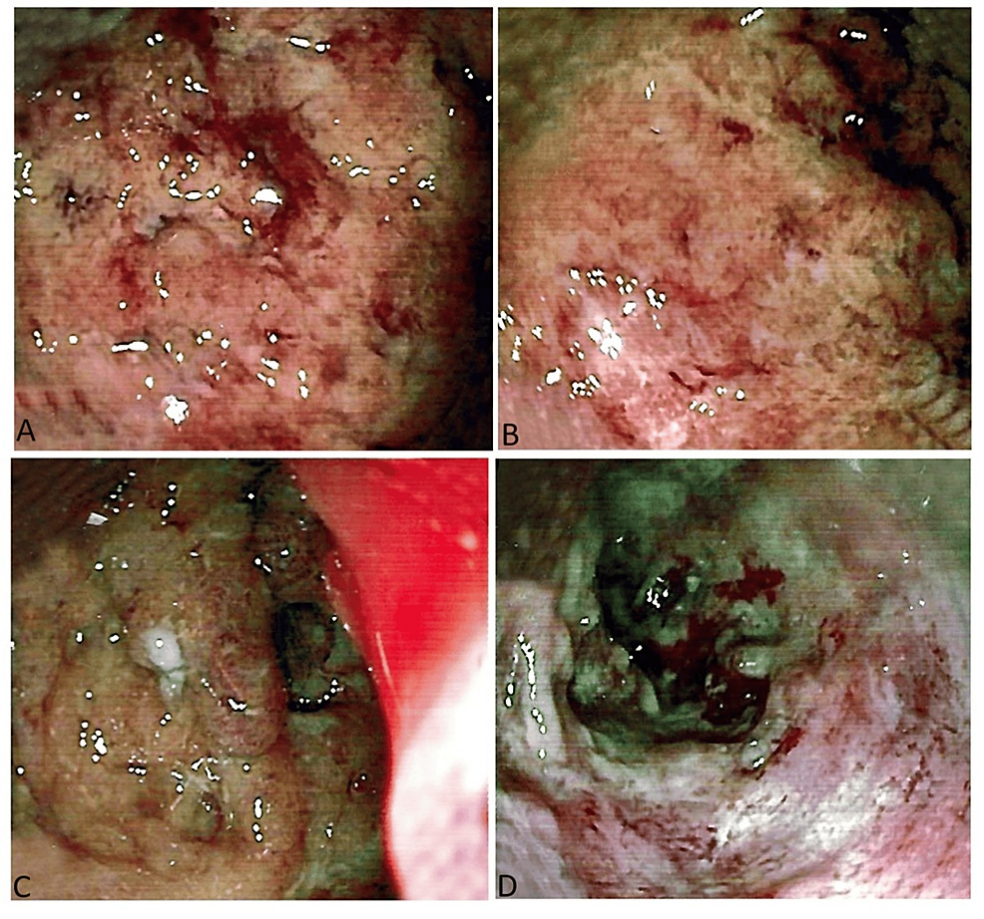
A, B) sigmoidoscopy of the patient showing active colitis with multiple deep ulceration in rectum; C, D) sigmid colon, Ulcerative Colitis Endoscopic Index of Severity score six

Sigmoidoscopy was performed with multiple biopsies taken for histopathological examination and CMV DNA polymerase chain reaction (PCR). As shown in Figure 2, findings indicated edematous mucosa with loss of vascularity, multiple deep ulcerations, and pseudopolypoid formations in the rectum, sigmoid, and descending colon, indicating an acute-on-chronic disease process. Tissue PCR confirmed a positive result for CMV, suggesting a superadded CMV infection. The patient's treatment plan was adjusted. Valganciclovir was started for the CMV infection. The patient stabilized, and discharge was planned with continued tofacitinib 10 mg once daily and Valganciclovir 450 mg twice daily for 30 days, with a follow-up scheduled for one month. After one month's follow-up, the patient did well without any major symptoms. Additionally, there was no pertinent family history of the patient related to this particular disease condition.

## Discussion

The presented case underscores the complexity of managing inflammatory bowel disease (IBD), particularly UC, when complicated by opportunistic infections. The patient, initially suspected to be experiencing an exacerbation of UC, was ultimately diagnosed with acute CMV colitis, highlighting the importance of considering infectious etiologies in the differential diagnosis of IBD flare-ups. The coexistence of UC and CMV colitis poses diagnostic challenges due to the overlap in clinical symptoms and endoscopic findings. Patients with UC often experience abdominal pain, diarrhea, and rectal bleeding during disease exacerbations, symptoms that can be mirrored in CMV colitis [6]. Endoscopic features such as mucosal edema, ulcerations, and pseudopolypoid formations in both conditions further complicate the distinction [7].

Immunosuppressive therapies used in managing UC, such as corticosteroids, biologics, and immunomodulators, can contribute to the reactivation of latent CMV [8]. In this case, the patient had a history of prior hospitalization for UC and had undergone treatment with intravenous infliximab, steroids, and later azathioprine. The combination of these immunosuppressive agents likely predisposed the patient to CMV reactivation, leading to the observed clinical presentation. The decision to discontinue azathioprine, infliximab, and initiate tofacitinib in the treatment plan was based on the need to balance immunosuppression with the management of the superimposed CMV infection. Tofacitinib, a Janus kinase (JAK) inhibitor, has shown efficacy in UC management and was chosen as an alternative immunomodulator to address the patient's symptoms while minimizing the risk of opportunistic infections [9].

Antiviral therapy with valganciclovir was initiated promptly to target the CMV infection. The successful outcome and clinical stabilization observed in this case supports the importance of early and accurate diagnosis of CMV colitis in patients with UC. This aligns with existing literature emphasizing distinguishing infectious complications from disease exacerbations to guide appropriate therapeutic interventions [10].

## Conclusions

In conclusion, this case report underscores the intricate nature of managing UC when complicated by opportunistic infections like CMV colitis, particularly in the context of immunosuppressive therapy. The initial clinical presentation mimicking a UC exacerbation highlights the diagnostic complexities faced by clinicians in distinguishing between disease flares and superimposed infections. The patient's history of UC and recent exposure to immunosuppressive agents played a crucial role in the reactivation of CMV, necessitating a careful reassessment of the treatment plan. The successful management of this case, involving the discontinuation of azathioprine and infliximab, initiation of tofacitinib, and antiviral therapy with Valganciclovir, exemplifies the need for a multidisciplinary approach and individualized treatment strategies. This case serves as a valuable reminder for clinicians to maintain a high index of suspicion for opportunistic infections in IBD patients, guiding timely interventions for improved clinical outcomes. Continued research efforts are essential to refine diagnostic approaches and therapeutic strategies in the nuanced landscape of inflammatory bowel disease complicated by infectious etiologies.
